# Reducing the aneuploid cell burden – cell competition and the ribosome connection

**DOI:** 10.1242/dmm.049673

**Published:** 2022-11-29

**Authors:** Nicholas E. Baker, Cristina Montagna

**Affiliations:** ^1^ Departments of Genetics, Developmental and Molecular Biology, and Ophthalmology and Visual Sciences, Albert Einstein College of Medicine, 1300 Morris Park Avenue, Bronx, NY 10461, USA; ^2^ Department of Radiation Oncology, Rutgers Cancer Institute of New Jersey, 195 Little Albany Street, New Brunswick, NJ 08901, USA

**Keywords:** Aging, Aneuploidy, Cancer, Cell competition, Ribosomal protein gene

## Abstract

Aneuploidy, the gain or loss of chromosomes, is the cause of birth defects and miscarriage and is almost ubiquitous in cancer cells. Mosaic aneuploidy causes cancer predisposition, as well as age-related disorders. Despite the cell-intrinsic mechanisms that prevent aneuploidy, sporadic aneuploid cells do arise in otherwise normal tissues. These aneuploid cells can differ from normal cells in the copy number of specific dose-sensitive genes, and may also experience proteotoxic stress associated with mismatched expression levels of many proteins. These differences may mark aneuploid cells for recognition and elimination. The ribosomal protein gene dose in aneuploid cells could be important because, in *Drosophila*, haploinsufficiency for these genes leads to elimination by the process of cell competition. Constitutive haploinsufficiency for human ribosomal protein genes causes Diamond Blackfan anemia, but it is not yet known whether ribosomal protein gene dose contributes to aneuploid cell elimination in mammals. In this Review, we discuss whether cell competition on the basis of ribosomal protein gene dose is a tumor suppressor mechanism, reducing the accumulation of aneuploid cells. We also discuss how this might relate to the tumor suppressor function of p53 and the p53-mediated elimination of aneuploid cells from murine embryos, and how cell competition defects could contribute to the cancer predisposition of Diamond Blackfan anemia.

## Teaser

**Summary:** We review evidence that aneuploid cells are eliminated in the presence of normal cells and the potential role of ribosomal protein gene haploinsufficiency in this cell competition process.

## Introduction

Aneuploidy is a product of genomic instability and is characterized by the gain or loss of entire chromosomes. Aneuploidy causes birth defects and miscarriages, and it is a distinctive feature of cancer, found in nearly all solid tumors and thought to contribute to cancer development. Chromosome instability (CIN), the genome instability process that drives aneuploidy, promotes tumor heterogeneity and drug resistance, altogether suggesting that aneuploidy itself contributes to tumor formation, as well as sustains tumor evolution. In addition to roles in cancer, recent reports associate aneuploidy with tissue degeneration (Andriani et al., 2016a; Macedo et al., 2018), wherein aneuploidy is proposed as a hallmark of aging because of the premature aging phenotypes associated with some CIN mouse models (Baker et al., 2006; Baker et al., 2004). Indeed, aneuploidy is sufficient to induce premature cellular senescence, a form of permanent cell cycle arrest that is a feature of aging (Andriani et al., 2016a; Crasta et al., 2012). Accordingly, mosaic aneuploidy has been shown to accumulate with age in the mammalian brain (Rehen et al., 2001; Pack et al., 2005; Yang et al., 2003; Yurov et al., 2005; Rehen et al., 2005; Kingsbury et al., 2005), liver (Duncan et al., 2010; Duncan et al., 2012a; Faggioli et al., 2008; Faggioli et al., 2011a), lymphocytes (Jacobs et al., 1961), vascular smooth muscle (Jones and Ravid, 2004) and oocytes (Jones, 2008). Finally, although aneuploidy is commonly associated with various pathologies, changes in ploidy also occur in mammals under physiological conditions, and it cannot be ruled out that they are sometimes adaptive.

Multiple safeguards exist within cells to limit the occurrence of aneuploidy, consistent with the idea that aneuploidy is generally detrimental. In this Review, we discuss the accumulating evidence that extrinsic defenses against aneuploidy also exist, so that even if aneuploidy arises in individual cells, these cells can be recognized and eliminated from the mosaic tissue. This process potentially suppresses tumorigenesis and promotes healthy aging, but how it occurs is insufficiently understood. We also explore the emerging evidence from *Drosophila* studies that aneuploid cells can be eliminated by cell competition based on altered ribosomal protein (Rp) gene dose, the circumstances in which this may occur, and the potential implications of this process for the origins and prevention of cancer, aging and other diseases.

## What is aneuploidy and where is it found?

Aneuploidy [‘an’ (not)+‘eu’ (well)+‘ploid’ (fold)] is a numerical alteration of whole-chromosome numbers. The deleterious consequences of an imbalanced (aneuploid) genome content on organism development were first described over a century ago by German zoologist Theodor Boveri while studying sea urchins (Boveri, 1914). He first recognized that aneuploidy is detrimental and has adverse consequences on cell and organism physiology.

**Fig. 1.**
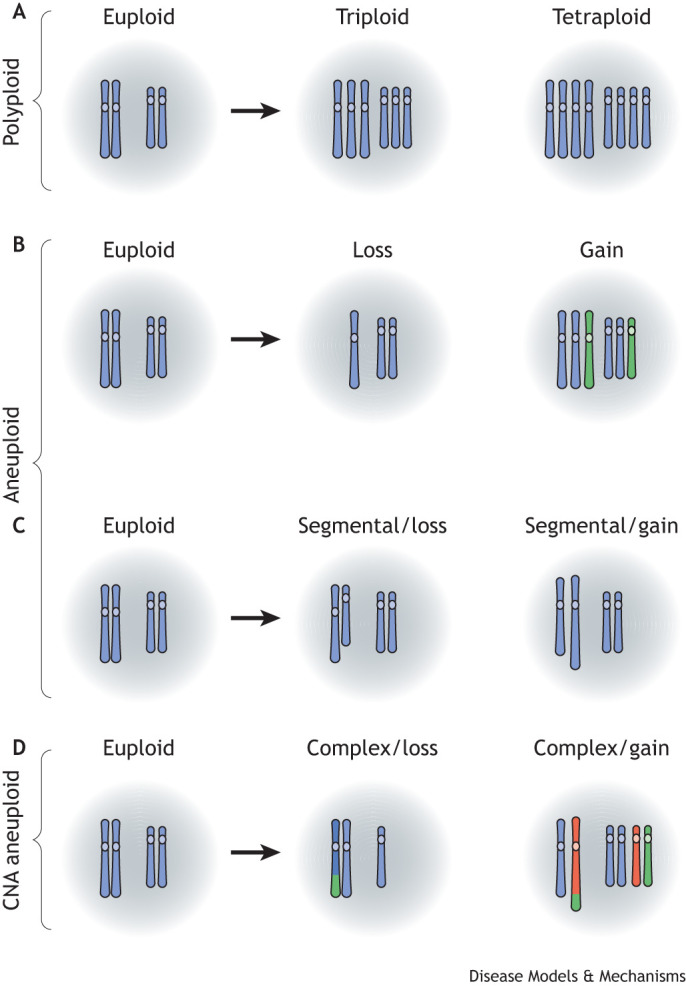
**Schematic representation of polyploidy and aneuploidy that can arise in mammalian cells.** (A) Polyploidy describes the condition in which cells obtain more than one copy of their entire chromosome complement. Because the gene ratios are maintained, this condition is not considered detrimental. (B,C) Aneuploidy is defined as the presence of unbalanced chromosome number, where either entire chromosome gains or losses (B) or segmental copy number changes that involve large chromosomal regions (C) can be observed. Because this disrupts gene ratios and, in the case of losses, reduces copy number of a large number of genes, aneuploidy is typically harmful. (D) An example of a complex karyotype that can arise as a consequence of unbalanced translocations in a euploid or polyploid cell. CNA, copy number alteration.

The simplest aneuploidies are monosomies, whereby the cell loses one chromosome of a diploid pair, and trisomies, in which cells with a diploid genome gain one extra chromosome (Fig. 1). In humans, whole-body monosomy of autosomes is incompatible with life, as is trisomy for most autosomes, resulting in miscarriage or newborn death. Accordingly, aneuploidy is a major cause of early pregnancy loss in humans (van den Berg et al., 2012), accounting for ∼50% of miscarriages in the first trimester (Palacios Jaraquemada, 2005). Trisomies 13, 18 and 21, and aneuploidies for the sex chromosomes are the exceptions. These are viable, with the affected individuals even reaching adulthood, albeit with developmental and cognitive abnormalities. Tumors typically exhibit complex karyotypes, with copy number alterations (CNAs) affecting many chromosomes, as discussed later in the Review. One can also refer to ‘segmental aneuploidy’, in which only a portion of a chromosome is monosomic or trisomic. Finally, it is worth mentioning that polyploidy, the duplication of the whole chromosome complement within the same cell, is not aneuploidy, because there is no mismatch of chromosome numbers, and gene dosage relationships are maintained. Indeed, it was the observation that polyploidy is less detrimental than chromosome imbalance that originally drew attention to the phenomenon of aneuploidy (Birchler and Veitia, 2021; Milan et al., 2014). Polyploidy is common in some human tissues under physiological conditions, in which it seems to be adaptive, not detrimental (Duncan et al., 2010; Banerjee and Wagner, 1972; Rios et al., 2016). However, aneuploidy can also arise in a polyploid background. Because chromosomal losses are better tolerated in a polyploid background, polyploidy may be a major route to sporadic aneuploidy *in vivo*.

Over the course of the past decade, it has become clear that the genome varies from cell to cell and that most, if not all, adult humans are genetic mosaics of postzygotic mutations (Jacobs et al., 2012; Costantino et al., 2021). Genetic mosaicism of different mammalian tissues has been reported for all types of mutations: single-nucleotide variants (Lodato et al., 2015; Lodato et al., 2018; Sun et al., 2022; Zhang et al., 2019), small insertions and deletions, copy number variation (CNV) (Cai et al., 2015; Zhang et al., 2009; McConnell et al., 2013), telomere instability (Brown et al., 2020), mitochondrial DNA CNV (Wachsmuth et al., 2016) and retrotransposition (Reilly et al., 2013; Evrony et al., 2012). Somatic whole-chromosome aneuploidy has been reported in various organs of mammals, and is better tolerated than whole-body aneuploidy, which is also known as germline and as constitutive aneuploidy. Rates of mosaic aneuploidy can be extraordinarily high in human embryos, occurring in as many as 70%. Aneuploidy in oocytes and spermatozoa is associated with infertility (Calogero et al., 2003; Nagaoka et al., 2012), and analysis of miscarriages in humans demonstrates that aneuploidy is associated with early pregnancy failure (Hassold et al., 1980), supporting the detrimental effects of a chromosome imbalance. The frequency of aneuploidy in somatic tissues remains unclear, with its occurrence presumed to be highly variable in different organs and age groups (Naylor and van Deursen, 2016).

Many mechanisms, extensively reviewed elsewhere, cause aneuploidy (Holland and Cleveland, 2012). One of the leading mechanisms is chromosome mis-segregation during mitosis. Interestingly, loss or gain of particular chromosomes may often follow cytokinesis failure that initially results in polyploidization. In line with this, aneuploid cells frequently display chromosome numbers that are near-tetraploid. Mosaic aneuploidy is more difficult to detect than constitutive aneuploidy, and estimates of its frequency may vary between tissue types but also depend on the methodologies used to detect it.

## Methodologies for measuring sporadic aneuploidy

**Fig. 2.**
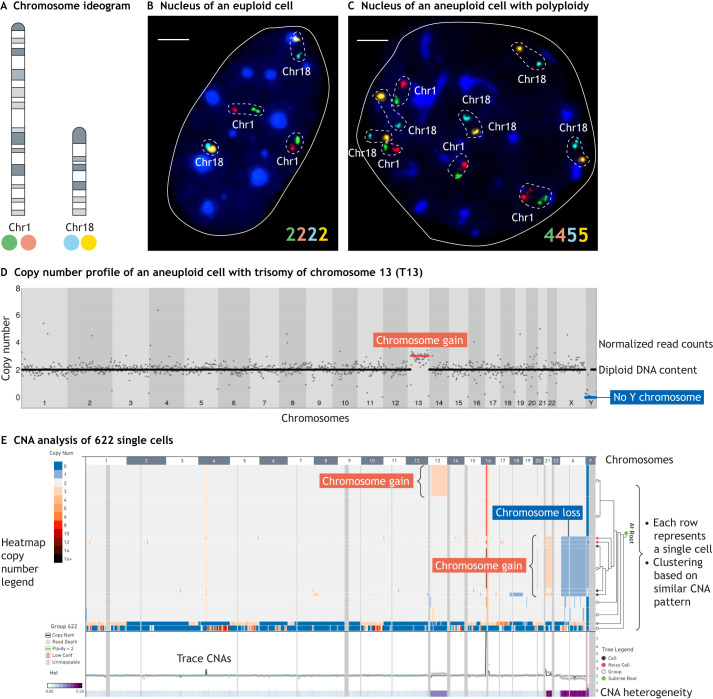
**Molecular cytogenetics and molecular genetics methodologies to detect somatic aneuploidy in cultured cells and in tissues.** (A-C) Two-color interphase FISH is a sensitive methodology to quantify chromosome copy number changes based on the presence of two probes mapping to different regions of the same chromosome. (A) Ideogram of mouse chromosomes 1 and 18 depicting the fluorophores used to label the corresponding locus-specific regions in this example. (B) Representative nucleus of a euploid cell in which two copies of chromosomes 1 and 18 are detected based on the number of locus-specific probe signals. (C) Representative image of an aneuploid nucleus in an otherwise polyploid background, in which four copies of chromosome 1 and five copies of chromosome 18 can be detected. In B and C, colored numbers on the bottom summarize the number of locus-specific signals for each chromosome-specific probe. Dashed lines highlight the two locus-specific signals mapping the same chromosome, and dark blue indicates total nuclear DNA [stained with the DNA dye 4′,6-diamidino-2-phenylindole (DAPI)]. Scale bars: 1 µm. Images acquired by Dr Francesca Faggioli in C.M.’s laboratory. (D,E) Aneuploidy detection based on ultra-low-coverage single-cell sequencing. Single cells or nuclei can be isolated from cell culture or tissues, and undergo whole-genome amplification followed by library construction and massively parallel sequencing using standard high-throughput methodologies. (D) Aligned sequencing reads can be ‘binned’ (grouped) in chromosomal regions of variable size, typically depicted as light and darker gray background and with chromosome numbers noted at the bottom. Normalized read counts can be used for the segmentation of CNAs across the entire genome, shown here as dots and the horizontal line. Therefore, the *y*-axis on this plot represents copy number values, and the *x*-axis shows autosomes and sex chromosomes. The plot shows a representative CNA profile of a cell with a gain of chromosome 13 (red horizontal line) and an XX sex chromosome profile, hence the lack of a Y chromosome (blue horizontal line). This plot was generated using the online bioinformatics tool Ginkgo (http://qb.cshl.edu/ginkgo/?q=/ieSGnzsRNikZIZr42WlY). (E) Summary view of an example heatmap depicting aneuploidy and large segmental CNAs. This heatmap shows 622 cells, with each row representing a single cell, and columns indicating autosomes and sex chromosomes. In this heatmap, warm colors (salmon and red) indicate copy number gains, and cold colors (shades of blue) indicate copy number losses. The analysis that produced the heatmap also allows the clustering of cells according to their CNA similarity. The ‘tree’ brackets on the right side of the heatmap highlight clusters of cells with similar CNAs (salmon indicating gains, blue indicating losses). The track at the bottom of the heatmap highlights CNA heterogeneity across the overall cell population (the 622 cells in this analysis): genomic regions with high variability of karyotype states are marked in dark purple, whereas light blue shows more stable genomic regions. This plot was generated using the 10X Genomics' Loupe browser (https://www.10xgenomics.com/products/loupe-browser). Chr, chromosome; CNA, copy number alteration; FISH, fluorescence *in situ* hybridization.

Investigating somatic mosaic aneuploidy is, unlike germline aneuploidy or clonally expanded aneuploidy in tumors, extremely challenging due to the stochastic nature of these events, which are unique to individual or a few cells (Fig. 2). Single-cell-based methods are essential, because bulk DNA analysis currently lacks the required sensitivity to reliably detect minor clone genotypes. Many studies, including those from our laboratories, have analyzed aneuploidy by fluorescent *in situ* hybridization (FISH) (Fig. 2A-C). Although FISH remains the gold standard for the detection of numerical chromosome changes in clinical settings, its application to the study of rare somatic aneuploidies is more challenging because the frequency of aneuploid cells can be small. For example, in solid adult tissues, the numerical change in gene dose is only around one to four copies, and different chromosomes are affected in different cells (Faggioli et al., 2011b; Westra et al., 2008; Rehen et al., 2001). A principal technical issue concerns distinguishing sporadic aneuploidy from artefacts due to FISH detection errors. Accordingly, studies applying FISH approaches have been criticized for overestimating the frequency of mosaic aneuploid cells (Knouse et al., 2014). FISH limitations can be overcome, in part, by custom assays such as interphase FISH (iFISH). iFISH significantly reduces the number of false positives by using multiple fluorescent probes marking separate regions on the same chromosome, which enhances sensitivity and specificity over the use of single probes (Fig. 2B,C) (Faggioli et al., 2014) (Faggioli et al., 2012).

More recently, single-cell sequencing methods to study aneuploidy have emerged (Fig. 2D,E). Although the advantage of surveying copy number changes across the entire chromosome complement is undeniable, these studies can lack power for accurate determination of CNA or aneuploidy when a limited number of cells are analyzed, usually 30-100, and from very few samples (McConnell et al., 2013; Knouse et al., 2014; Cai et al., 2014). Because single-cell isolation and library preparation costs limit the throughput, it is not yet practical or economic to match the number of cells that can be analyzed by iFISH, which can reach the order of thousands per sample. Most importantly, low-coverage single-cell whole-genome sequencing, commonly adopted to study aneuploidy and CNA, is also limited by the whole-genome amplification methods needed to prepare single-cell sequencing libraries. These limitations result in a global underestimation of aneuploidy, especially when it occurs as complex aneuploidy in a polyploid background (Andriani et al., 2019).

These technological issues may explain the wide range of somatic aneuploid frequencies reported for adult tissues. The brain, an organ of exceptional complexity, has been the focus of several studies to describe somatic mosaic changes of DNA content during development, aging and disease, but these ultimately reported highly discrepant findings. Some identified somatic aneuploidy with a frequency as high as 30% (Faggioli et al., 2012; Pack et al., 2005; Yurov et al., 2009; Iourov et al., 2009; Arendt et al., 2010; Fischer et al., 2012; McConnell et al., 2013; Rehen et al., 2001; Yang et al., 2003; Westra et al., 2008; Andriani et al., 2016b; Peterson et al., 2012), whereas others reported absence (van den Bos et al., 2016; Knouse et al., 2014) or extremely low levels (∼1%) (van den Bos et al., 2016; Knouse et al., 2014). Thus, there has been intense debate over the degree of aneuploidy in neurons and other brain cell types. Emerging methodologies and analytical tools for the detection of aneuploidy and large CNAs from single-cell RNA-sequencing data may resolve these differences in the future (Delaney, 2021). Given the different outcomes of FISH- and single-cell-sequencing-based approaches, arguments can be made that the true frequency of aneuploidy in the brain is ∼10% (Toda et al., 2019). Regardless of the exact frequency, aneuploidy could have a profound impact on brain physiology and functions, possibly with regional and cell-type-specific differences.

## What are the main effects of aneuploidy?

Two main genetic consequences of aneuploidy are often discussed in the literature. One is that aneuploidy changes the relative copy number of genes on the affected chromosomes, for example proto-oncogenes and tumor suppressors, resulting in chromosome-specific effects on the affected cells (Padilla-Nash et al., 2013; Ried et al., 2012; Upender et al., 2004). This may be responsible, in part, for preferential chromosome gains and losses in cancer, which are, to some extent, tissue specific (Table 1). The second consequence, described in a seminal study in yeast, was that cells carrying extra copies of one or more chromosomes shared common phenotypes that were independent of the specific chromosome(s) affected, and that these phenotypes resemble a stress response (Torres et al., 2007a). These include reduced proliferation, reduced viability, and sensitivity to defects in protein synthesis and folding (Torres et al., 2007b; Terhorst et al., 2020). These cell responses have been attributed to the stress of stoichiometric mismatches between individual components of protein complexes. That is, because many cellular proteins function partly or entirely in complexes, mismatches in their expression levels due to differences in copy number of their respective genes are expected to yield orphan, unassembled proteins, which may either be degraded or participate in abnormal functions. The cumulative effect of hundreds of such mismatches in aneuploid cells is proposed to be the cause of the aforementioned aneuploidy stress. Importantly, in mammals, gene dose affects the expression of most genes (Upender et al., 2004; Mao et al., 2003), as was long ago recognized for lower organisms (Ciferri et al., 1969; Lucchesi and Rawls, 1973), even though protein expression levels may be compensated more than mRNA expression levels (Stingele et al., 2012; Schukken and Sheltzer, 2022). This means that aneuploidy potentially results in genetic variation, as well as a proteotoxic and metabolic stress common to all aneuploidies (Santaguida and Amon, 2015; Santaguida et al., 2017; Santaguida et al., 2015).

**Table 1.**
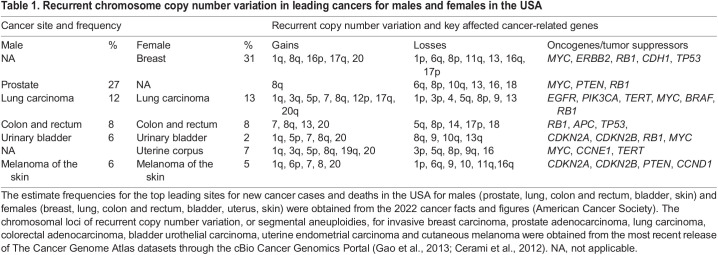
Recurrent chromosome copy number variation in leading cancers for males and females in the USA

Perhaps related to the distinct effects that they cause, not all true aneuploidies function in the same way. In culture, primary human telomerase reverse transcriptase (hTERT)-immortalized human retinal pigment epithelial cells that are aneuploid for multiple chromosomes arrest immediately upon chromosome mis-segregation, whereas cells that contain aneuploidy affecting less than 5% of their genomes can sustain cell division despite the abnormal chromosome number (Santaguida et al., 2017). Accordingly, *in vivo*, high aneuploidy levels carry a putative disadvantage for clonal expansion, whereas low aneuploidy levels may provide a selective advantage (Weaver et al., 2007).

As mentioned above, polyploidy, which is more common in some human tissues, must be distinguished from aneuploidy, because it does not change the relative gene dose of proto-oncogenes and tumor suppressors, or result in proteotoxic stress. For example, the high numbers of binucleated alveolar cells that arise as consequence of cytokinesis failure in the mammary glands of mice, humans, seals and wallabies during the late stages of pregnancy, are not detrimental, but have been shown to have essential roles for lactogenesis (Rios et al., 2016; Smith, 2016). This mechanism presumably evolved to sustain the high-energy requirement for milk production while maintaining gene expression levels that are proportional, in general, to gene number. Polyploidy is also common in the liver, where over half of the mature hepatocytes in mice and humans are also aneuploid, at least 25% of which are also aneuploid (Duncan et al., 2010; Faggioli et al., 2011a; Duncan et al., 2012b). It has been proposed that this has evolved as an adaptive mechanism to xenobiotic or nutritional injury (Duncan et al., 2012a).

It is additionally important to distinguish simple aneuploidy, caused by rare mis-segregation of one or a few chromosomes, from CIN, the propensity of the genome to undergo multiple chromosome gains and losses. CIN promotes the continuous evolution of karyotypes and therefore should be defined as the rate of karyotype evolution, whereas aneuploidy defines a cell with an abnormal chromosome content. CIN resulting in aneuploidy is sufficient to induce senescence, a stable cell cycle arrest linked to aging and other diseases (Campisi, 2011). Aneuploid cells can acquire a senescence-associated secretory phenotype characterized by the secretion of IL1B, CXCL8, CCL2, TNF, CCL27 and other pro-inflammatory factors (Andriani et al., 2016a; He et al., 2018; Macedo et al., 2018) (Wang et al., 2021b), some of which may even be general indicators of the cells' aneuploid state. Because aneuploidy promotes further genomic instability by increasing DNA damage and replication stress (Passerini et al., 2016), it can be regarded as a general indicator of genomic instability and may be one of the hallmarks of aging (Faggioli et al., 2011b). Indeed, age-related accumulation of aneuploidy has been reported in peripheral B-lymphocytes (Forsberg et al., 2014; Thompson et al., 2019; Pierre and Hoagland, 1972), the brain (Faggioli et al., 2012; Andriani et al., 2016b; Iourov et al., 2021), the ovaries (Antico Arciuch et al., 2011) the vascular endothelium (Aviv et al., 2001), germinal cells (Sloter et al., 2004) and the eye (Matsuyama et al., 2013).

The detrimental effects of aneuploidy also make it a plausible cause of disease (Faggioli et al., 2011b; Andriani et al., 2017). The evidence obtained particularly supports roles for aneuploidy in neurodegenerative disorders. Mosaic aneuploidy in Alzheimer's disease was first proposed in 1991 (Potter, 1991) and extensively investigated with complementary molecular approaches (Yang et al., 2001; Kingsbury et al., 2006; Mosch et al., 2007; Thomas and Fenech, 2008; Iourov et al., 2009), all reporting high levels of aneuploidy (>30%) in the neurons of patients. Somatic mosaic aneuploidy can also be detected in cells from patients' peripheral tissues, including fibroblasts and peripheral blood mononuclear cells (Mukherjee and Thomas, 1997), and buccal cells (Jacobs et al., 2012). Nearly all Down's syndrome individuals older than 50 years of age display plaques and tangles within their brains that are similar in form, number and distribution to those observed in Alzheimer's disease patients (Mann and Esiri, 1988). This is possibly a consequence of the overexpression of the amyloid precursor protein gene that maps to chromosome 21.

Mosaic aneuploidy for chromosomes 18 and X appear to occur at higher frequency in Alzheimer's disease patients than in controls (Geller and Potter, 1999; Yurov et al., 2014). Increased CIN has been reported in patients with the neurological disorder ataxia telangiectasia, where the levels of aneuploid neurons were threefold higher relative to those in age- and sex-matched controls (Iourov et al., 2009; Yurov et al., 2009). Aneuploidy and cellular senescence are also linked to eye diseases (Garcia-Castillo et al., 2008; Matsuyama et al., 2013; Grassmann et al., 2020).

It has also been argued that the genetic diversity due to somatic aneuploidy in the mammalian brain, where aneuploid neurons can be functionally active and part of the normal organization, might be adaptive. Here, euploid and aneuploid neurons can form brain circuits with unique signaling properties (Kingsbury et al., 2005; Westra et al., 2008). In support of some beneficial effects of aneuploidy beyond the nervous system, research has shown that aneuploidy can confer a selective advantage to human cells grown under conditions of environmental stress (Rutledge et al., 2016; Ippolito et al., 2021). Overall, extensive experimental evidence supports detrimental consequences of aneuploidy under physiological conditions, but it cannot be ruled out that, in some conditions, aneuploidy may promote genetic diversity and be adaptive.

## Aneuploidy and cancer

It remains to be explained why aneuploidy seems not to be detrimental to cancer cells. In fact, cancer aneuploidy is so frequent as to suggest that aneuploidy is a requirement for malignancy (Table 1). The causal effect of aneuploidy in cancer is supported by several experimental models and the extensive molecular characterization of human tumors of any epithelial origin. Most of the chromosomally unstable mouse models generated to date are cancer prone (Pfau and Amon, 2012). Genetic alterations that promote CIN potentiate tumorigenesis, possibly through loss of whole chromosomes containing tumor suppressor genes (Baker et al., 2009; Baker and van Deursen, 2010), and even specific single chromosome aneuplodies can be tumor promoting in humans and mice (Ben-David et al., 2014; Rutledge et al., 2016). Conversely, protection against aneuploidization in transgenic mice by sustained high expression of the core mitotic checkpoint protein BubR1 (BUB1), which suppresses chromosome mis-segregation and thus prevents aneuploidy, significantly reduced susceptibility to spontaneous and carcinogen-induced lung and skin tumors (Baker et al., 2013). In humans, biallelic mutations in the cell division regulators BUB1B (Matsuura et al., 2006), CEP57 (Snape et al., 2011) or TRIP13 (Yost et al., 2017) cause mosaic variegated aneuploidy, a rare syndrome in which some cells in the body have an abnormal number of chromosomes and that renders affected individuals more susceptible to cancer (Hanks et al., 2004).

The prevalence of somatic CNA in tumors indicates that ∼25% of the cancer cell genome contains whole-arm or whole-chromosome aneuploidy (Beroukhim et al., 2010). These show preferential gains or losses, which suggests a positive selection pressure for CNA of particular genes. Aneuploidy has been implicated not only in cancer formation and growth, but also in the metastatic process. Cancer cells in metastases at distant sites acquire specific karyotypes distinct from those of the primary tumor, which again suggests that dosage of particular genes is relevant. Thus, CIN could promote genetic diversity and facilitate the emergence of cancer cell populations with a genetic makeup suitable for metastasis (Gao et al., 2016).

The cancer predisposition of mouse models of CIN, as well as of individuals with mosaic variegated aneuploidy, suggests, but does not prove, that aneuploidy is an early contribution to cancer initiation. Notably, even when murine models of human cancer are induced by targeted alteration of classical human oncogenes or tumor suppressor genes that does not involve promoting aneuploidy, the resulting tumors are highly aneuploid with patterns of chromosomal gains and losses similar to those observed in human cancer (Ried et al., 2004; Wang et al., 2021a). Thus, bypassing aneuploidy as a potential requirement for cancer initiation by directly manipulating key cancer-causing genes does not prevent the eventual appearance of aneuploidy.

Thus, although aneuploidy is mainly detrimental in physiological conditions, it is a defining feature in cancer. As it already occurs in premalignant lesions, this supports the notion that it is a driver of malignant transformation. Aneuploidy could be an attractive target for cancer therapy or prevention (Cohen-Sharir et al., 2021). The pattern of genomic imbalances is specific to organ site, and to some extent to defined stages of tumor development. How some aneuploid cells are tolerated, if and how they escape surveillance mechanisms, and how they contribute to transformation, remain unknown.

## Cell-extrinsic defense against aneuploid cells

If aneuploidy is generally detrimental and promotes tumorigenesis, one would expect that organisms might have evolved adaptive processes to remove individual aneuploid cells or cells with other extensive genetic damage. Evidence that such damaged cells can be removed comes from mammalian embryos, in which mosaic aneuploidy is very common (Hook, 1981; van Echten-Arends et al., 2011; Bazrgar et al., 2013; Greco et al., 2015). Yet the majority of mosaic embryos develop into normal babies with no evidence of aneuploidy-associated birth defects, suggesting that there is a mechanism that facilitates the elimination of abnormal cells during embryonic development (Hook, 1981; van Echten-Arends et al., 2011; Bazrgar et al., 2013; Greco et al., 2015).

Selective loss of aneuploid cells has been replicated and modeled in mouse embryogenesis by treating embryonal stem cells with reversine, an inhibitor of the spindle assembly checkpoint (SAC) that typically increases aneuploidy. Whereas mouse embryos reconstituted using reversine-treated stem cells develop abnormally, this was not the case in mouse embryos reconstituted by mixtures of reversine-treated and of untreated control cells, which developed into normal adult mice with no apparent phenotypic contribution of the reversine-treated cells (Bolton et al., 2016). Further work has shown that the aneuploid cells in these embryos are removed by a p53 (TP53)-dependent cell death mechanism that occurs specifically in the presence of the normal cells (Singla et al., 2020). Observations of aneuploid cell frequencies that vary over time also support a process of targeted elimination. For example, the mouse cortex is estimated to contain 30% aneuploid cells at embryonic day 13.5, but these decrease to 1% by 4 months post-partum (Faggioli et al., 2012; Andriani et al., 2016b). In this case, however, it is not known whether aneuploid cell loss is stimulated by the presence of many normal euploid cells within the same tissue or whether it represents a cell-autonomous process, i.e. reduced grow rate, that would occur in the aneuploid cell regardless of the presence of euploid neighboring cells.

**Fig. 3.**
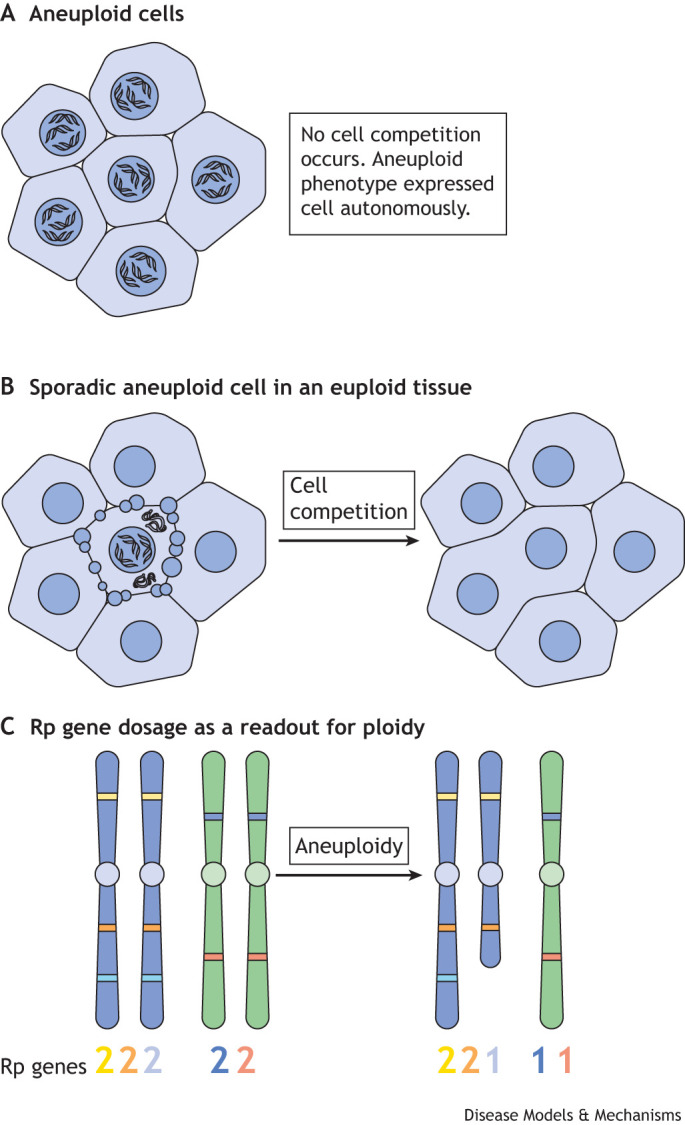
**Cell competition: removal and replacement of aneuploid cells.** Aneuploidy can have both cell-intrinsic and -extrinsic consequences. (A) Aneuploid cells can experience cell-intrinsic stress due to mismatched protein levels, leading to defects in cell cycle progression and sensitivity to protein folding. This stress response occurs independently of other cells and has even been observed in the unicellular yeast *Saccharomyces cerevisiae* (Torres et al., 2007a). (B) Sporadic aneuploid cells surrounded by normal, euploid cells may be eliminated by cell competition, whereby having euploid neighbors triggers defects in proliferation and survival (Bolton et al., 2016; Ji et al., 2021). Notably, cell competition was initially recognized in *Drosophila* tissues as a non-autonomous effect on survival of cells that carried heterozygous point mutations in ribosomal protein (Rp) genes. These heterozygous (Rp-deficient) cells are replaced by their wild-type neighbors over time (Baker, 2020). (C) Eukaryotic genomes encode 80 Rp genes, which are scattered through the genome, occupying loci on almost all chromosomes. Owing to this distribution, most aneuploid genotypes result in altered Rp gene dose. This means that Rp quantification can potentially be used as a surrogate readout for distinguishing aneuploid from euploid cells. In this figure, segmental monosomy of the blue chromosome reduces the dose of the cyan Rp locus relative to the yellow and orange Rp loci, and whole-chromosome monosomy of the green chromosome results in reduced doses of the blue and red Rp loci.

Specific removal implies that aneuploid cells differ physiologically from euploid cells and can be recognized as such. Unlike cells in which chemically altered DNA initiates DNA damage responses, the chemical structure of DNA in aneuploid cells is intact, meaning that mechanisms capable of recognizing only the relative amounts of the different chromosomes must exist. As noted above, aneuploidy results in proteotoxic stress due to mismatched protein levels (Fig. 3A), but it remains to be fully explained how such cell-autonomous consequences of aneuploidy could lead to the recognition and eventual elimination of aneuploid cells in mosaics containing normal euploid cells (Fig. 3B) (Santaguida et al., 2017). The remainder of this Review focuses on the possible contribution of cell competition to removing aneuploid cells.

## Rp gene dose and aneuploidy

The argument has been made that Rp genes in particular play a key role in the elimination of aneuploid cells (McNamee and Brodsky, 2009). Rps are highly expressed and required in stoichiometrically equal amounts. Orphan Rps, which are not assembled into ribosomes, are rapidly degraded (Abovich et al., 1985; Lam et al., 2007). In yeast, orphan Rps can aggregate (Albert et al., 2019; Tye et al., 2019). Because Rp genes are generally single copy, and their mapping is spread around the eukaryote genome (Uechi et al., 2001), they can act as reporters for the copy number of whole chromosomes or chromosome segments (Fig. 3C). Changes in Rp gene dose were therefore hypothesized to contribute significantly to the cellular stress responses observed in aneuploidy (McNamee and Brodsky, 2009; Baker, 2011). Remarkably, this idea has received strong support from studies of human cells (Chunduri et al., 2021). Cell lines with monosomies for individual chromosomes, derived from immortalized human retinal pigmented epithelium cells, share a gene expression profile that downregulates ribosomal subunit assembly and translation. This led to the conclusion that monosomy generally impairs ribosome biogenesis and translation due to the presence of one or more RP loci on almost every human chromosome, and that this impaired translation is distinct from the stress response first described for yeast with extra chromosomes (Chunduri et al., 2021).

The role of Rp genes in *Drosophila* has been of particular interest, partly because this organism is apt for genetic studies and has contributed much to studies of aneuploidy (Milan et al., 2014; Birchler and Veitia, 2021), but also because it is well known that *Drosophila* cells with heterozygous Rp point mutations are subject to elimination via the phenomenon of cell competition (Morata and Ripoll, 1975; Simpson, 1979; Baker, 2020; Morata, 2021). Almost 100 years ago, drosophilists in the renowned ‘fly room’ of T. H. Morgan's laboratory identified numerous mutant *Drosophila* strains with dominant developmental retardation and small body bristle size, along with other defects (Bridges and Morgan, 1923; Lambertsson, 1998). This phenotype, named ‘Minute’ after the small bristles, was much later found to reflect haploinsufficiencies for ∼65 of the 79 Rp genes (Kongsuwan et al., 1985; Marygold et al., 2007). While investigating the cell autonomy of these mutations in *Drosophila* tissues, Morata and Ripoll, and later Simpson, observed not only that the growth of Minute clones in mosaic tissues was retarded, just as in whole-body Rp-mutant Minute flies, but that Minute cells were subjected to progressive and selective elimination in the presence of wild-type cells. This elimination did not occur in non-mosaic tissues in which all cells were Minute (Morata and Ripoll, 1975; Simpson, 1979). It is now known that Minute cells are eliminated via apoptosis, which occurs selectively near the interfaces between wild-type and Minute cells (Moreno et al., 2002; Li and Baker, 2007), much like the elimination of aneuploid cells from mosaic mammalian embryos that we discussed above (Bolton et al., 2016; Singla et al., 2020). Thus, to the extent that aneuploidy changes Rp gene dose, elimination of aneuploid cells might occur by a mechanism similar to that already observed when Rp gene dose is reduced by point mutations (Fig. 3C).

**Fig. 4.**
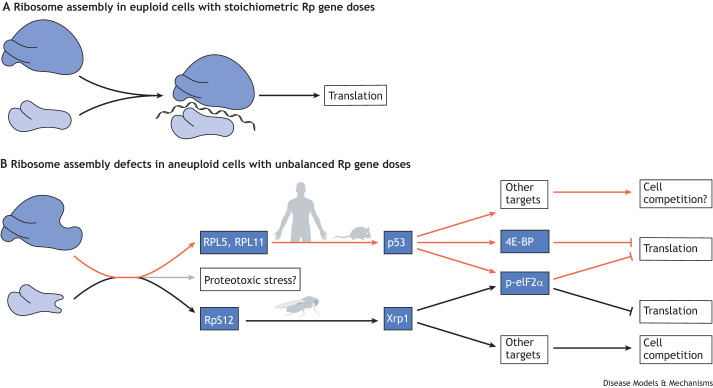
**Ribosome protein stoichiometry and ribosome assembly stress.** In euploid cells, stoichiometric amounts of ribosomal RNAs and proteins assemble into 40S small and 60S large ribosome subunits. These subunits assemble along with mRNA into 80S monosomes that are competent for translation. A shortage of a protein component of either the large or small subunit results in the accumulation of intermediates and orphan ribosome components, which affects cellular regulatory pathways (Ferreira-Cerca et al., 2005; Kiparaki et al., 2022). In mammalian cells, relative excess of the 5S ribonucleoproteins RPL5 and RPL11 stabilizes p53, which has many cellular consequences. One of these may be a reduction in global translation, which has been reported to occur due to eIF2α phosphorylation, phosphorylation of eEF2 or enhanced transcription of 4E-BP (EIF4EBP), in cells carrying mutations in different Rp genes (Tiu et al., 2021; Solanki et al., 2016; Knight et al., 2021). p53 affects many other targets and is commonly implicated in mammalian cell competition, although whether cell competition affects mammalian cells with heterozygous mutations in Rp genes remains to be demonstrated directly (Baker, 2020). In *Drosophila*, a relative excess of the RpS12 protein promotes the expression of the transcription factor Xrp1, resulting in reduced global translation due to eIF2α phosphorylation, as well as transcriptional changes thought to promote cell competition (Kiparaki et al., 2022; Kale et al., 2018; Ji et al., 2019; Lee et al., 2018; Ochi et al., 2021). p-, phosphorylated.

The molecular mechanisms by which Minute cells are eliminated from mosaic tissues in *Drosophila* are beginning to be uncovered, which helps to assess whether cell competition is indeed important for removing aneuploid cells (Fig. 3B). First, as mentioned above, cell competition relies on apoptosis pathways (Moreno et al., 2002; Tyler et al., 2007; Kale et al., 2015; Martin et al., 2009). Second, cell competition occurs between Rp-mutant and wild-type cells; because the latter are a requirement, cell competition does not occur between two populations of Rp-mutant cells, even between populations that carry mutations in distinct Rp loci. This means that a whole-body mutation in an Rp locus permits the survival of clones that carry additional mutations in any other Rp gene (Simpson, 1979). Third, cell competition depends on Xrp1, a bZip-domain transcription factor that controls a transcriptional stress response in Rp-mutant cells (Baillon et al., 2018; Lee et al., 2018; Lee et al., 2016). Xrp1 is not completely specific for cell competition. Although it seems to have little or no function in normal flies, it is a target of p53 in the DNA damage response (Brodsky et al., 2004; Akdemir et al., 2007), acts in certain neurological pathologies (Mallik et al., 2018) and may play a role in P element transposition (Francis et al., 2016). During cell competition, Xrp1 expression is activated by RpS12, a specific Rp that seems to report defects in ribosome assembly (Fig. 4) (Lee et al., 2018; Ji et al., 2019). It is not known whether RpS12 affects the translation of *Xrp1* mRNA or acts through another mechanism, but cell competition and Xrp1 induction are prevented by a particular *rpS12* point mutation that has no major effects on growth or viability (Ji et al., 2019; Kale et al., 2018). It has also been proposed that ribosome biogenesis defects lead to the aggregation of orphan Rp and hence to proteotoxic stress that then contributes to cell competition (Baumgartner et al., 2021; Recasens-Alvarez et al., 2021). Rp aggregates have not been demonstrated directly in *Drosophila*, but Rp aggregation in yeast leads to a transcriptional stress response through the RASTR pathway, not a general proteotoxic stress (Albert et al., 2019; Tye et al., 2019).

Based on these findings, it has been possible to evaluate whether the cell competition mechanism removes aneuploid cells on the basis of Rp gene dose (Ji et al., 2021), a model that was first supported by studies of DNA damage (Titen and Golic, 2008; McNamee and Brodsky, 2009). Using molecularly defined excisions of chromosome regions to generate segmentally aneuploid cells within tissues derived from *Drosophila* imaginal discs, our own group demonstrated that heterozygous deletions affecting hundreds of genes were generally compatible with apparently normal proliferation and differentiation, so long as the chromosomal excisions did not affect any Rp genes. However, when we excised Rp loci, this led to the exclusion of the affected cells from adult tissues. Importantly, these segmentally aneuploid cells were eliminated by cell competition with euploid cells and not by cell-autonomous defects, because they survived and differentiated when the entire tissue was aneuploid (Ji et al., 2021). Segmentally aneuploid cells also survived and differentiated into apparently normal organs if they carried a background mutation in another Rp gene inherited from the germline, so that the wild-type cells necessary for cell competition were lacking. Additionally, the same study showed that survival of segmentally aneuploid cells was enhanced by inactivating mutations in components of the apoptosis pathway, or in *Xrp1*, or affecting the RpS12-dependent induction of Xrp1. These results were consistent with the genetically defined pathway of Minute cell competition being required for the elimination of cells with aneuploidy-spanning Rp loci. Finally, restoration of a diploid dose of the *RpL28* gene rescued cells heterozygous for a 3.18 Mb deletion in the third chromosome that includes this gene, confirming that the correlation between Rp gene dose and cell fate in mosaics reflected a causal role for the Rp genes (Ji et al., 2021). Although this study was restricted to segmental aneuploidies, not whole-chromosome aneuploidies, it should be noted that *Drosophila* has only three pairs of autosomes, one of which is very small. Gain or loss of major *Drosophila* chromosomes is genetically comparable to the gain or loss of ∼10 human chromosomes, thus resembling the highly aneuploid genotypes occurring in human cancer, whereas segmental aneuploidy in *Drosophila* is comparable to the gain or loss of single human chromosomes. In addition, the study did not address trisomies, because extra copies of Rp genes do not seem to be deleterious or to promote cell competition (Tyler et al., 2007; Meyer et al., 2014; Baillon et al., 2018). Some other mechanism might be needed to eliminate trisomic cells, if such an elimination occurs. Another consideration is that cell competition has not been observed in all tissues. The fly abdomen is an example of an adult tissue in which cell competition is not observed, and, perhaps, in consequence, the fly abdomen is more tolerant of aneuploid cells (Ripoll, 1980). Whether limitations also apply to the elimination of aneuploid cells in mammals, or whether additional mechanisms apply in mammalian tissues, remains to be established.

## Aneuploidy and proteotoxic stress

Because our group sought to establish the contribution of Rp loci in *Drosophila*, we focused on segmental aneuploidies that include no to three *Rp* loci, not on whole-chromosome aneuploidies. It is not yet known whether cell competition is also responsible for eliminating cells with larger genetic changes akin to highly aneuploid human cancer cells.

*Drosophila* cells with more extensive aneuploidies, including increases in chromosome number, have been generated by inhibition of the SAC (Dekanty et al., 2012; Benhra et al., 2018). The SAC prevents chromosome mis-segregation by blocking chromosome separation in the metaphase–anaphase transition until chromosomes are properly aligned and attached to the mitotic spindle. Impairing the *Drosophila* SAC by mutation or knockdown of genes encoding constituent proteins results in extensive aneuploidy. Most of the aneuploid cells that survived such SAC inhibition had chromosome numbers above diploid (Dekanty et al., 2012). This is in accordance with previous conclusions that whole-chromosome trisomies are tolerated in *Drosophila*, whereas whole-chromosome monosomy is only tolerated if it affects the small fourth chromosome (Ashburner et al., 2005). It is generally thought that, unlike segmentally aneuploid cells, the more highly aneuploid cells generated by SAC inhibition are eliminated regardless of the genotype of their neighbors, i.e. by cell-autonomous mechanisms rather than by cell competition, although this has not been established directly (Dekanty et al., 2012). These highly aneuploid cells do exhibit proteotoxic stress, similar to that seen in yeast cells with trisomies (Torres et al., 2007a), and their viability can be enhanced by genetic rescue of proteotoxic stress factors. One of the key outcomes of proteotoxic stress appears to be mitochondrial dysfunction. This occurs when autophagy is close to saturation because of the extent of proteotoxicity, causing defects in mitophagy-mediated mitochondrial recycling (Joy et al., 2021; Benhra et al., 2018).

A second difference between segmentally aneuploid cells and cells with defective chromosome segregation occurs when apoptosis is prevented. Blocking apoptosis in highly aneuploid cells allows them to delaminate from the epithelium and develop into tumors characterized by invasive overgrowth (Dekanty et al., 2012), whereas preventing apoptosis of segmentally aneuploid cells does not produce tumors (Ji et al., 2021).

These two differences in *Drosophila* could reflect differences in the degree of aneuploidy, which affects ∼5% of the genome, or less in cells with segmental aneuploidies, but causes larger genomic changes in cells exposed to SAC inhibition. Alternatively, they could represent differences between monosomy and trisomy, or other complex aneuploidies, as suggested by the gene expression profiles of aneuploid human cells (Chunduri et al., 2021).

## Does cell competition affect mammalian aneuploidy and cancer?

The *Drosophila* studies discussed above have revealed a potential role for Rp genes as sensors that lead to the selective elimination of aneuploid cells (Fig. 3). These cells are detected due to their defects in ribosome assembly (Fig. 4A,B) and eliminated in a cell non-autonomous manner. Conversely, aneuploidies that lead to a general proteotoxic stress result in cell death that might occur in a cell-autonomous manner, independently of cell competition. Does cell competition affect aneuploid cells in human tissues, where aneuploidy contributes to aging and cancer?

As noted above, cell competition only removes Rp-haploinsufficient cells in the presence of wild-type ones, so *Drosophila* tissues in which all cells are Rp haploinsufficient are unable to remove segmentally aneuploid cells by cell competition (Ji et al., 2021). If this inability to remove cells is conserved, it suggests that cell competition does not occur in humans suffering from Diamond Blackfan anemia (DBA), the majority of whom are heterozygous non-mosaic for mutations in any of a number of RP loci (Ulirsch et al., 2018). Intriguingly, DBA patients experience a 4.8× increase in their lifetime risk of developing cancer, apparently without regard to tissue (Vlachos et al., 2018; Vlachos et al., 2012). Could DBA patients be predisposed to cancer because aneuploid cells accumulate in their tissues but are not eliminated by cell competition? If this were indeed the explanation, it would suggest that almost 80% of human pre-neoplasms are normally eliminated by cell competition on the basis of aneuploidy. It would remain to be explained how some aneuploid cells escape cell competition to form cancers in normal individuals. However, other explanations for why RP mutations might promote tumorigenesis have also been suggested, so their potential impact on cell competition and on aneuploid cell burden is only one of several hypotheses that have been discussed elsewhere (Sulima et al., 2019; Sulima et al., 2017).

A key unanswered question is whether RP mutations are tumor promoting in a cell-autonomous manner, denoting an oncogenic effect on cells themselves, or in a non-autonomous manner, preventing aneuploid cells from being recognized as different and eliminated by cell competition. Certain RP loci are commonly mutated within tumors themselves, supporting cell-autonomous oncogenic effects of these mutations (Derenzini et al., 2019). It remains to be seen whether a non-autonomous contribution also exists.

5q syndrome, a myelodysplastic disorder that often progresses to malignancy, is associated with mosaic heterozygosity of a chromosome region that includes the *RPS14* gene. The occurrence of this syndrome indicates that clones of *RPS14*-heterozygous cells can survive in the bone marrow (Ebert et al., 2008; Vlachos, 2017). This challenges the idea that RP-heterozygous cells are always competitively disadvantaged in humans. In contrast to *RPS14*, however, *Rpl24*-mutant cells demonstrably experience a growth disadvantage in chimeric mice (Oliver et al., 2004). Moreover, reversing *RPS19*, *RPL4*, *RPS26* or *RPL41* haploinsufficiencies in DBA patient bone marrow leads to remission, which is associated with the clonal expansion of healthy RP wild-type cells and suggests that cells with these RP haploinsufficiencies experience growth disadvantages in the bone marrow (Garelli et al., 2019; Jongmans et al., 2018; Venugopal et al., 2017). If *RPS14* deletion in 5q syndrome represents an exceptional RP mutant genotype that survives in mosaic tissues, perhaps this could be influenced by neighboring loci that are also affected by the 5q deletions, rather than being solely due to *RPS14* haploinsufficiency itself.

It has yet to be determined whether Rp-heterozygous cells are eliminated via cell competition in mammals. This may occur, however, because heterozygous cells experience a growth disadvantage in chimeric mice (Oliver et al., 2004) and because wild-type cells seem to replace Rp-heterozygous ones in the bone marrow when DBA reverts (Oliver et al., 2004; Garelli et al., 2019; Jongmans et al., 2018; Venugopal et al., 2017). Despite these effects, competitive cell interactions have not yet been demonstrated, and could be caused simply by cell-autonomous growth-repressing effects of Rp mutations. It is worth mentioning that even cell-autonomous growth deficits could reduce the burden of aneuploid cells.

As already established, human cancer cells often have highly abnormal karyotypes. These cells likely experience proteotoxic stress beyond that caused by RP gene dose imbalances. As we previously discussed, in *Drosophila*, highly aneuploid genotypes are tumorigenic when they are prevented from undergoing cell death (Dekanty et al., 2012). However, little is known about how human cancer cells acquire their highly abnormal karyotypes. If highly aneuploid cells develop from cells with fewer initial abnormalities, perhaps cell competition might select against these more deeply aneuploid cells at the earlier stages of tumorigenesis. Consequently, the potential contributions of RP gene dose and cell competition to tumor surveillance depend on these little-known aspects of aneuploidy development in tumors.

The transcription factor Xrp1, which plays a key role in competition of *Drosophila* cells with Rp mutations (Fig. 4B), lacks a close mammalian ortholog (Blanco et al., 2020). Interestingly, this is not because Xrp1 has been dispensable during evolution, but because Xrp1 is under very strong positive selection for rapid evolutionary change, for example, as though it is targeted by pathogens with which it is in an evolutionary arms race (Blanco et al., 2020). It has been suggested that some of the functions of Xrp1 in *Drosophila* might be performed by mammalian DDIT3 (CHOP), a similar bZip protein (Blanco et al., 2020), and some by p53. This latter suggestion is because Xrp1 is a transcriptional target of *Drosophila* p53 in the DNA damage response, and because p53 has been implicated in many examples of cell competition in mammals (Baker et al., 2019). Perhaps the p53-dependent resolution of mosaic aneuploidy in mouse embryos we discuss above (Singla et al., 2020) is a further example. Interestingly, mammalian Rp mutations lead to p53 activation, which is responsible for significant aspects of the phenotype, possibly even the overall reduction in translation (Fig. 4B) (Tiu et al., 2021). Accordingly, it has been suggested that, in *Drosophila*, the effects of Rp mutations, including cell competition, are mediated by the p53 target Xrp1, whereas in mammals, these mutations result in the activation of p53 itself (Baker et al., 2019).

Could cell competition be related to the tumor-suppressor function of p53? Intriguingly, cancers containing monosomies are more likely to lack p53 function, suggesting a selection against the p53 activity caused by RP haploinsufficiency (Chunduri et al., 2021). p53 is known as the guardian of the genome, coordinating transcriptional responses to DNA damage. These include arrest of the cell cycle to facilitate DNA repair and apoptosis to remove more severely damaged cells, yet it is increasingly uncertain whether these functions are sufficient to explain the importance of p53 as a tumor suppressor (Kaiser and Attardi, 2018). Studies of conditional loss of p53 function in mice establish that the tumor suppressor role of p53 is not contemporary with the acute response to DNA damage, but occurs later, after DNA repair and DNA damage-induced apoptosis have occurred (Christophorou et al., 2006; Christophorou et al., 2005; Hinkal et al., 2009). Other genetic changes that prevent either cell cycle arrest in response to p53 or p53-dependent apoptosis also fail to prevent p53 function as a tumor suppressor (Brady et al., 2011; Jiang et al., 2011; Li et al., 2016; Efeyan et al., 2006).

If p53 suppresses tumorigenesis by facilitating competitive elimination of aneuploid cells, then p53 mutations might contribute to sporadic aneuploid cells escaping competitive elimination. However, in DBA patients, who have increased risk of developing cancer, p53 mutations might not be necessary for tumorigenesis if cell competition is defective. Other models for the tumor predisposition of DBA patients make the opposite prediction. For example, if chronic p53 activity in DBA patient cells, which suppresses their proliferation, creates a growth advantage for p53-mutant cells and the accumulation of mutant cells within tissues then facilitates tumorigenesis, this would predict that the majority of DBA patient tumors should be p53 defective. The same is true if p53 is protective against a cell-autonomous, oncogenic effect of RP mutations. It would be interesting, therefore, to establish whether tumors in DBA patients are less frequently mutated for p53 than site-matched tumors in otherwise normal individuals.

## Conclusions

We have reviewed mosaic aneuploidy in otherwise normal tissues and the consequences of aneuploidy for individual cells. Aneuploidy is probably detrimental in most cases, and the evidence is very strong that aneuploidy contributes both to the origin and development of malignant tumors. Accordingly, specific elimination of aneuploid cells from mosaic tissues is likely to be tumor suppressive and promote healthy aging generally. A new hypothesis for aneuploid cell elimination is that it relates to altered Rp gene dose, because Rp-haploinsufficient cells are eliminated from mosaic *Drosophila* tissues by cell competition. Cell competition depends on a transcriptional response to Rp mutation in *Drosophila* cells, which may resemble the activation of p53 that occurs in mammalian cells with Rp mutations.

There are many outstanding questions regarding the molecular mechanisms of cell competition and their control by p53 and other factors. For example, even in *Drosophila*, the organism in which cell competition is arguably best understood, it remains uncertain what exactly is the difference between the cell surfaces of euploid and aneuploid cells that allows their selective detection and elimination. Experimental evidence suggests a number of hypotheses, and this remains a topic of active investigation (Moreno et al., 2002; Rhiner et al., 2010; Kucinski et al., 2017; Nagata et al., 2019; Ochi et al., 2021; Baker, 2020; Kiparaki et al., 2022). Uncovering these mechanisms promises new insights into the origin and development of tumors, aging and other processes related to genome damage.
